# Using time-series chest radiographs and laboratory data by machine learning for identifying pulmonary infection and colonization of *Acinetobacter baumannii*

**DOI:** 10.1186/s12931-023-02624-x

**Published:** 2024-01-03

**Authors:** Zhaodong Zeng, Jiefang Wu, Genggeng Qin, Dong Yu, Zilong He, Weixiong Zeng, Hao Zhou, Jiongbin Lin, Laiyu Liu, Chunxia Qi, Weiguo Chen

**Affiliations:** 1 Department of Radiology, NanFang Hospital of Southern Medical University, Guangzhou, China; 2 Department of Respiratory and Critical Care Medicine, Chronic Airways Diseases Laboratory, Nanfang Hospital of Southern Medical University, Guangzhou, China; 3 Department of Hospital Infection Management, ZhuJiang Hospital of Southern Medical University, Guangzhou, China; 4 Department of Hospital Infection Management, NanFang Hospital of Southern Medical University, Guangzhou, China

**Keywords:** *Acinetobacter baumannii*, Machine learning, Time-series chest radiographs and laboratory data, Infection and colonization

## Abstract

**Background:**

Accurately distinguishing between pulmonary infection and colonization in patients with *Acinetobacter baumannii* is of utmost importance to optimize treatment and prevent antibiotic abuse or inadequate therapy. An efficient automated sorting tool could prompt individualized interventions and enhance overall patient outcomes. This study aims to develop a robust machine learning classification model using a combination of time-series chest radiographs and laboratory data to accurately classify pulmonary status caused by *Acinetobacter baumannii*.

**Methods:**

We proposed nested logistic regression models based on different time-series data to automatically classify the pulmonary status of patients with *Acinetobacter baumannii.* Advanced features were extracted from the time-series data of hospitalized patients, encompassing dynamic pneumonia indicators observed on chest radiographs and laboratory indicator values recorded at three specific time points.

**Results:**

Data of 152 patients with *Acinetobacter baumannii* cultured from sputum or alveolar lavage fluid were retrospectively analyzed. Our model with multiple time-series data demonstrated a higher performance of AUC (0.850, with a 95% confidence interval of [0.638–0.873]), an accuracy of 0.761, a sensitivity of 0.833. The model, which only incorporated a single time point feature, achieved an AUC of 0.741. The influential model variables included difference in the chest radiograph pneumonia score.

**Conclusion:**

Dynamic assessment of time-series chest radiographs and laboratory data using machine learning allowed for accurate classification of colonization and infection with *Acinetobacter baumannii.* This demonstrates the potential to help clinicians provide individualized treatment through early detection.

**Supplementary Information:**

The online version contains supplementary material available at 10.1186/s12931-023-02624-x.

## Background

*Acinetobacter baumannii* (*A. baumannii*) is a significant nosocomial pathogen and a growing public health concern [1]. It has emerged as a frequent causative agent of lower respiratory tract infections in critically ill patients, known for its rapid acquisition of drug resistance and pan-drug-resistant phenotypes [2–4]. Strains of this bacterial species can cause lower respiratory tract infection while also colonizing the region asymptomatically [5–8]. Identifying the presence of colonization and infection is crucial for guiding initial antibiotic therapy and the implementation of isolation measures in order to prevent transmission of *A. baumannii* [9, 10]. Nevertheless, it is challenging to distinguish between the two. It is not always clear whether the patient is being colonized or infected at any given time, particularly in those who are immunosuppressed or at high risk, such as transplant patients, those with malignancies, or those receiving corticosteroids, owing to a suppressed inflammatory response [11].

Previous published studies have employed retrospective or prospective research methods to analyze the risk factors associated with colonization or infection [12–16]. Most studies indicated that parameters associated with infection by *A. baumannii* are the admission at ICU, the number of days of hospitalization, mechanical ventilation and antibiotic treatment. However, previous published studies have only analyzed data based on single-time clinical information when the strain was initially cultured. However, some clinical indicators of the hospitalized patients changed during this period, such as inflammatory indicators [17]. Dynamic vital laboratory data can provide valuable insights beyond individual data points. In addition, continuous observation of chest radiographs reflects changes in inflammation in the lungs [18]. Leveraging the full potential of these dynamic factors was expected to be advantageous for distinguishing between infection and colonization. There is a demand for a more objective, efficient, and intelligent approach to handling this time-dependent dataset with a linear relationship.

In recent years, the use of machine learning in healthcare has garnered increasing attention, particularly in areas such as lesion prediction, personalized patient treatment, and objective evaluation of patient conditions [19–21]. For instance, Wang at al. proposed an innovative Lasso Logistic Regression model that utilizes feature-based time series data to determine the optimal timing for drug administration or escalating intervention procedures in COVID-19 patients [19]. However, there have been limited studies focusing specifically on the application of machine learning in assessing the pulmonary status of *A. baumannii*. Therefore, the objective of this study is to effectively leverage the extensive data collected from various tests conducted during patient hospitalization to develop a machine learning classification model capable of accurately identifying *A. baumannii* pulmonary status. Additionally, we sought to extract key features from the time-series profiles of chest radiographs and laboratory data that significantly influenced the progression of infection.

## Methods

### Study design

**Fig. 1:**
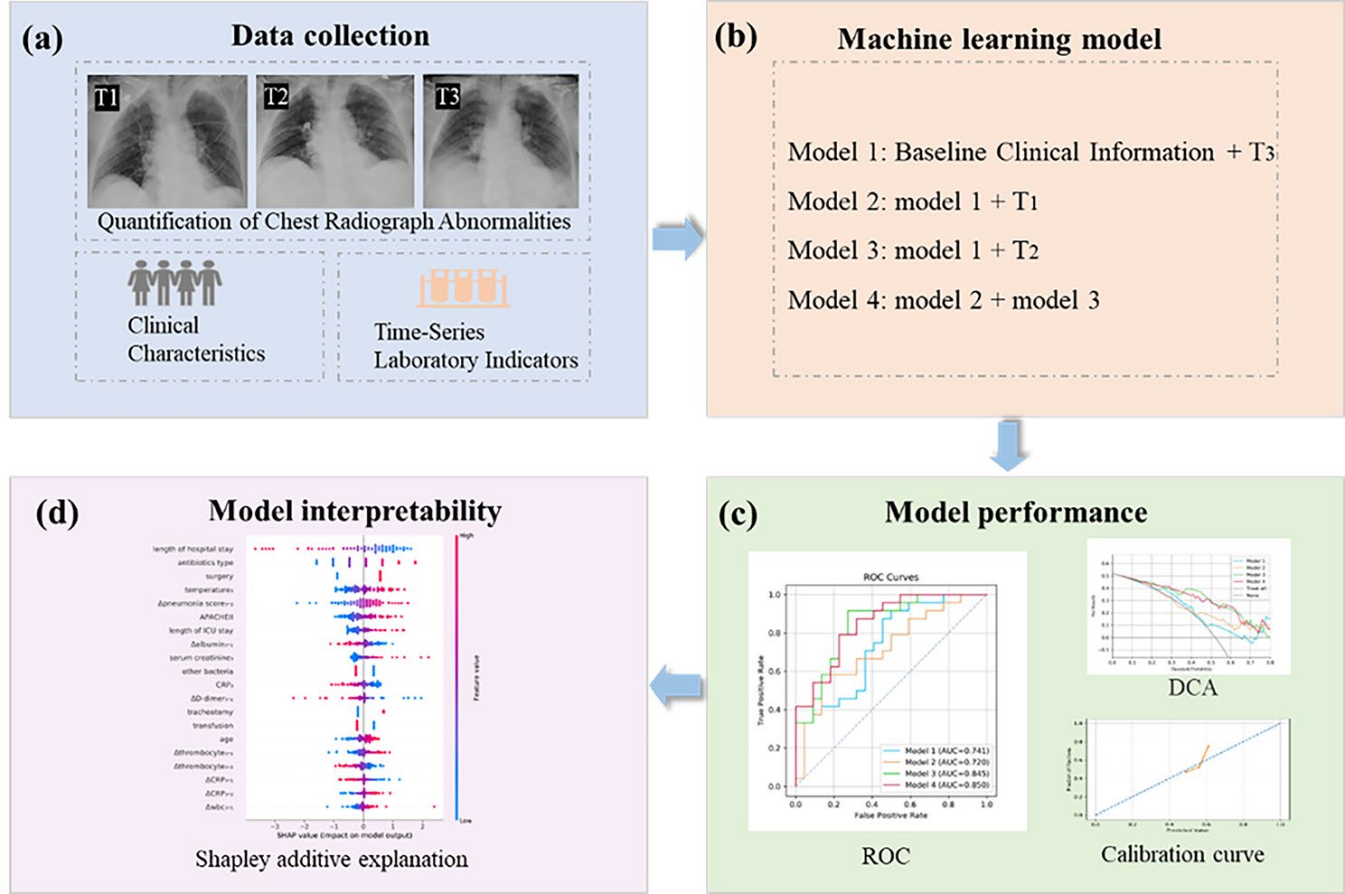
The overall design of the study. (**a**) Retrospective collection of baseline clinical information as well as time-series chest radiographs and laboratory indicators. Quantitative assessment of chest radiographs abnormalities, specifically through the quantitative scoring of chest radiographs by radiologists, aiming to detect and diagnose pneumonia more accurately. **(b)** We constructed four nested logistic regression models for classifying pulmonary infection and colonization of *A. baumannii* combined difference clinical characteristic. **(c)** Model performance was assessed using AUC, decision curve analysis and calibration curve. **(d)** We adopted Shapley additive explanation (SHAP) values to determine which features contributed most to model predictions on the logistic regression predictions. T_1_: within 1 day of admission; T_2_, 3 days before culturing out the strain; T_3_, 1 day within culturing out the strain. Model 1: clinical baseline information + laboratory indicators and radiographic features of T_3_. Model 2: model1 + the change value of between T_3_ and T_1_. Model 3: model 1 + the change value of between T_3_ and T_2_. Model 4: model 2 + model 3. ROC: receiver operating characteristic curves. DCA: decision curve analysis

Figure 1 depicts the study design. In this retrospective study, we examined the initial clinical characteristics, laboratory data, and chest radiograph data of patients with *A. baumannii* isolated from sputum or alveolar lavage fluid on more than two consecutive occasions. The study received approval from the Ethics Committee of Nanfang Hospital, and the need for informed consent was waived given the retrospective nature of the study. This study was conducted in compliance with the Transparent Reporting of a Multivariable Prediction Model for Individual Prognosis or Diagnosis (TRIPOD) guidelines [22].

### Study participants and data collection

We retrospectively enrolled 152 patients with *A. baumannii* cultured in the sputum or alveolar lavage fluid more than two consecutive times. Diagnoses of *A. baumannii* infection or colonization were confirmed by infectious disease specialists based on “Consensus of the Chinese specialists for diagnosis, treatment & control of *Acinetobacter baumannii* infection.” [23].

Patients with lower respiratory tract chest abnormalities resulting from noninfectious causes such as pulmonary embolism, pulmonary edema, lung cancer, and other conditions were excluded. Patients whose strain culture was performed within 3 days of hospitalization were also excluded. Chest radiographs were obtained from these patients within 1 day of *A. baumannii* culture. All conventional radiographic images were obtained using the hospital’s picture archiving and communication system. Baseline clinical information and chest radiographs were collected to create a dedicated database.

Data related to various categories were collected for analysis. This included baseline clinical information such as demographic characteristics (age and sex), preoperative comorbidities, invasive procedures, use of Antibiotics and glucocorticoids, hospitalization details, and time-series data. The time-series data consisted of three phases: T_1_ (within 1 day of admission), T_2_ (3 days before strain culture), and T_3_ (within 1 day of strain culture). Time-series data included chest radiographs and laboratory test results for serum inflammatory indicators, liver function, kidney function, and electrolytes. The baseline data used for the analysis were obtained at T_3_. To compare changes over time, the difference between the baseline data and the other two time points was calculated for each time-series datapoint. Additionally, the Acute Physiology and Chronic Health Evaluation (APACHE) II score was calculated within 1 day of strain isolation. More detail sees supplemental material.

### Imaging analysis: quantification of chest radiograph abnormalities

Chest radiographs were acquired through both portable and nonportable imaging devices, using both anteroposterior and posteroanterior projections. All time-series chest radiographs were independently and simultaneously reviewed by two experienced radiologists (J. Wu and J. Lin, with 9 and 15 years of diagnostic imaging experience, respectively) who were blinded to the clinical characteristics, laboratory findings, and patient outcomes. After independent evaluation, the radiologists resolved any disagreements with discussion and consensus. Serial chest radiographs were analyzed and compared to evaluate the progression, stability, or improvement of lung abnormalities throughout the course of the illness. For each patient diagnosed with pneumonia, the predominant chest radiographic features, as defined by the Fleischner Society Glossary [24] included ground glass opacity (GGO), consolidation, pleural thickening, adhesions, and pleural effusion. Additional information regarding the image interpretation process is available in the supplementary materials.

The severity score for each lung was calculated using the Radiographic Assessment of Lung Edema (RALE) score, as proposed by Warren et al. [25]. To quantitatively assess the extent of pulmonary abnormalities, such as ground-glass opacities (GGO) and consolidation, a chest radiograph score was assigned based on the involvement area in each of the six lung fields. The scoring criteria were as follows: 0 for no involvement, 1 for < 5% involvement, 2 for 5–25% involvement, 3 for 25–50% involvement, 4 for 50–75% involvement, and 5 for > 75% involvement. Additionally, a small amount of pleural effusion was given a score of 1, while a large pleural effusion and pleural thickening adhesions were assigned a score of 2. The total pneumonia severity score was calculated by summing up the scores of individual lung fields, resulting in a total score ranging from 0 to 34.

### Machine learning model development and evaluation

*Classification method*. For model development, we used logistic regression, a machine learning algorithm. Logistic regression diagnostics assess the effectiveness of models in capturing the underlying associations between predictors and patient outcomes in the given dataset, whether it is the dataset used for building the model or data from a distinct population [26]. The models were trained using Python (Python Software Foundation, version 3.7.4). The hyperparameters of the models were tuned using grid searching (for logistic regression) with cross-validation of the training set. To prevent overfitting and reduce model complexity, we first implemented the Least Absolute Shrinkage and Selection Operator (LASSO) to filter the features. The filtered features were then applied to the model for training. Missing values for continuous variables such as APACHE II were imputed using mean value.

*Comparison models.* To evaluate the additive value of dynamic changes in laboratory data and chest radiograph characteristics for classification ability, we generated the following nested logistic regression models that added different input variables to the training set: Model 1 (baseline model), clinical baseline information + laboratory data, and radiographic features at T_3_; Model 2, Model 1 + the change in value between T_3_ and T_1_; Model 3, Model 1 + the change in value between T_3_ and T_2_; Model 4 (multiple time-series model), Model 2 + Model 3.

*Calibration and decision curve analysis.* we assessed prediction performance by computing the net benefit through decision curve analysis (DCA) [27]. DCA integrates important insights into the advantages of accurately prioritizing patients (true positives) and the potential risks of excessive prioritization (false positives), ultimately, presenting a net benefit across a range of threshold probabilities for the outcome (or clinical preference). Furthermore, to enhance usability, we performed score recalibration using a sigmoid function on cross-validation samples and evaluated the probability of pulmonary *A. baumannii* classification.

*Feature importance.* To improve the interpretability of our model, we adopted Shapley Additive Explanation (SHAP) [28] values to determine which features contributed the most to model predictions in the logistic regression predictions. The SHAP value measures the contribution of each feature to the assigned infection risk level, either positively or negatively, as determined by the model. We employed an open-source implementation of the SHAP value method for both calculation and visualization purposes.

### Statistical analysis

Continuous variables were compared using Student’s t-test, while categorical values were compared using the χ^2^ test to identify any significant differences between the infection and colonization groups. Mean and standard deviation (SD) or median and interquartile range (IQR) were used to present continuous variables, while categorical variables were expressed as frequency (proportion). The area under the curve (AUC) was utilized as a comprehensive measure of discrimination, and the non-parametric Delong method was used to compare AUCs. To evaluate the model, we employed the bootstrap method to sample 1000 different test sets and obtain a 95% confidence interval (95%CI) for the model evaluation metrics. Additionally, accuracy, sensitivity, and specificity of different models were calculated. All statistical analyses were performed using MedCalc® statistical software (version 20.2; 2011 MedCalc Software bvba, Mariakerke, Belgium). A *p*-value of < 0.05 was considered statistically significant.

## Result

### Patient characteristics

A total of 152 patients met the inclusion criteria and were included in the study. Among these patients, 80 were infected and 72 were colonized by the strain of interest. The 152 patients were randomly divided into a training set of 106 patients and a test set of 46 patients. Table 1 summarizes the demographic, clinical, and time-series characteristics of the infection and colonization groups. The average age of the infection and colonization groups was 62(IQR, 53–74 years) and 59 years (IQR, 51–70 years), respectively. Notably, hypoproteinemia, cerebrovascular disease, combined fungal, APACHE II scores, and types of antibiotics used differed significantly between the colonization and infection groups (*p* < 0.05). Particularly, the APACHE II scores in the infection group were higher than those in the colonization group (median: 14[IQR, 10–19] vs. median: 12[IQR, 8–16], *p* = 0.006). Moreover, more types of antibiotics were used in the infection group prior to the strain being cultured than in the colonization group (median: 4[IQR, 2–5] vs. median: 3[IQR, 2–4], *p* = 0.031).

**Table 1:** Comparison of Demographic, Clinical, Time-series Laboratory data and Imaging Characteristics between Groups

Characteristic	Infection (n = 80)	Colonization (n = 72)	*Ρ* value
Age (y), median (IQR)	66(53–74)	62(51–70)	0.173
Comorbidities, n (%)			
Hypertension	37(46.3)	30(41.7)	0.570
Hypoproteinemia	48(60.0)	31(43.1)	**0.037**
Respiratory failure	39(37.5)	19(26.4)	0.143
Cerebrovascular disease	12(15.0)	3(4.2)	**0.025**
Combined fungal, n (%)	23(28.7)	8(11.1)	**0.007**
Length of hospital stay (d)	14.29 ± 0.97	16.44 ± 1.36	0.192
Length of ICU stay (d)	11.03 ± 1.11	9.28 ± 0.85	0.214
Duration of Oxygen supply (d) ^ƚ^			
Endotracheal intubation	6.00 ± 0.71	6.47 ± 0.97	0.691
Tracheotomy	3.18 ± 0.97	2.57 ± 0.76	0.631
Nasal catheter	2.08 ± 0.47	1.74 ± 0.63	0.663
Types of drugs, median (IQR) ^ƚ^			
Glucocorticoid	2(1–2)	2(1–2)	0.208
Antibiotics	4(2–5)	3(2–4)	**0.031**
APACHEII, median (IQR)	14(10–19)	12(8–16)	**0.006**
Temperature_3_(℃)	37.64 ± 0.10	37.28 ± 0.09	**0.009**
Laboratory results			
Percentage of neutrophils^§^ (%)	81.18 ± 1.07	81.41 ± 1.08	0.880
CRP (mg/L) ^§^	80.99 ± 6.17	77.33 ± 7.17	0.702
D-dimer (mg/mL) ^§^	7.03 ± 0.87	8.94 ± 1.53	0.260
PCT (ng/ml) ^§^	1.77 ± 0.46	1.20 ± 0.23	0.307
ΔCRP_3 − 2_	3.27 ± 7.37	16.56 ± 11.00	0.124
ΔWBC_3 − 2_ (×10^9^/L)	1.92 ± 1.00	-0.55 ± 0.79	0.055
ΔD-dimer _3−2_	0.10 ± 0.93	-1.63 ± 1.65	0.348
ΔSerum creatinine _3−2_(µmol/L)	10.81 ± 7.84	-13.45 ± 9.08	**0.044**
ΔPCT_3 − 1_	-5.15 ± 2.83	-4.37 ± 3.32	0.860
ΔD-dimer _3−1_(µg/mL)	-2.48 ± 1.75	1.20 ± 2.40	0.207
ΔPercentage of neutrophils_3 − 1_	3.63 ± 1.83	7.47 ± 2.63	0.224
Pneumonia scores, median (IQR)			
Pneumonia scores _3_^§^	15(10-21.5)	13(9–19)	0.108
ΔPneumonia scores _3−1_	5(0–9)	2(-3-8.5)	0.131
Δpneumonia scores _3−2_	1(-2.75-4)	-2(-5.75-1)	**0.001**

Note. Except where indicated, data are means ± SDs. ^ƚ^ Indicates the current period of hospitalisation. ^§^ Baseline data is the data within 1 day of culturing out the strain. The symbol “Δ” represents the value of change between the data at different time points. Δvalue _3−2_ represents the change value of the time-series feature between T_3_ and T_2_. Δvalue _3−1_ represents the change value of the time-series feature between T_3_ and T_1_. Bolded indicates statistically significant differences. CRP = C-reactive protein level, PCT = procalcitonin, WBC = white blood cell

Regarding the time-series data, Δserum creatinine _3−2_ and Δpneumonia scores_3 − 2_ exhibited significant differences between groups (*p* = 0.044). Patients with infection in the pulmonary had Δpneumonia scores_3 − 2_ statistically higher than the colonization group (*p* = 0.001). More notably, the pneumonia score of the infected patients increased, while that of the colonized group decreased from T_2_ to T_3_. The remaining time-series characteristics were balanced between the two groups.

### Model performance assessments

**Fig. 2:**
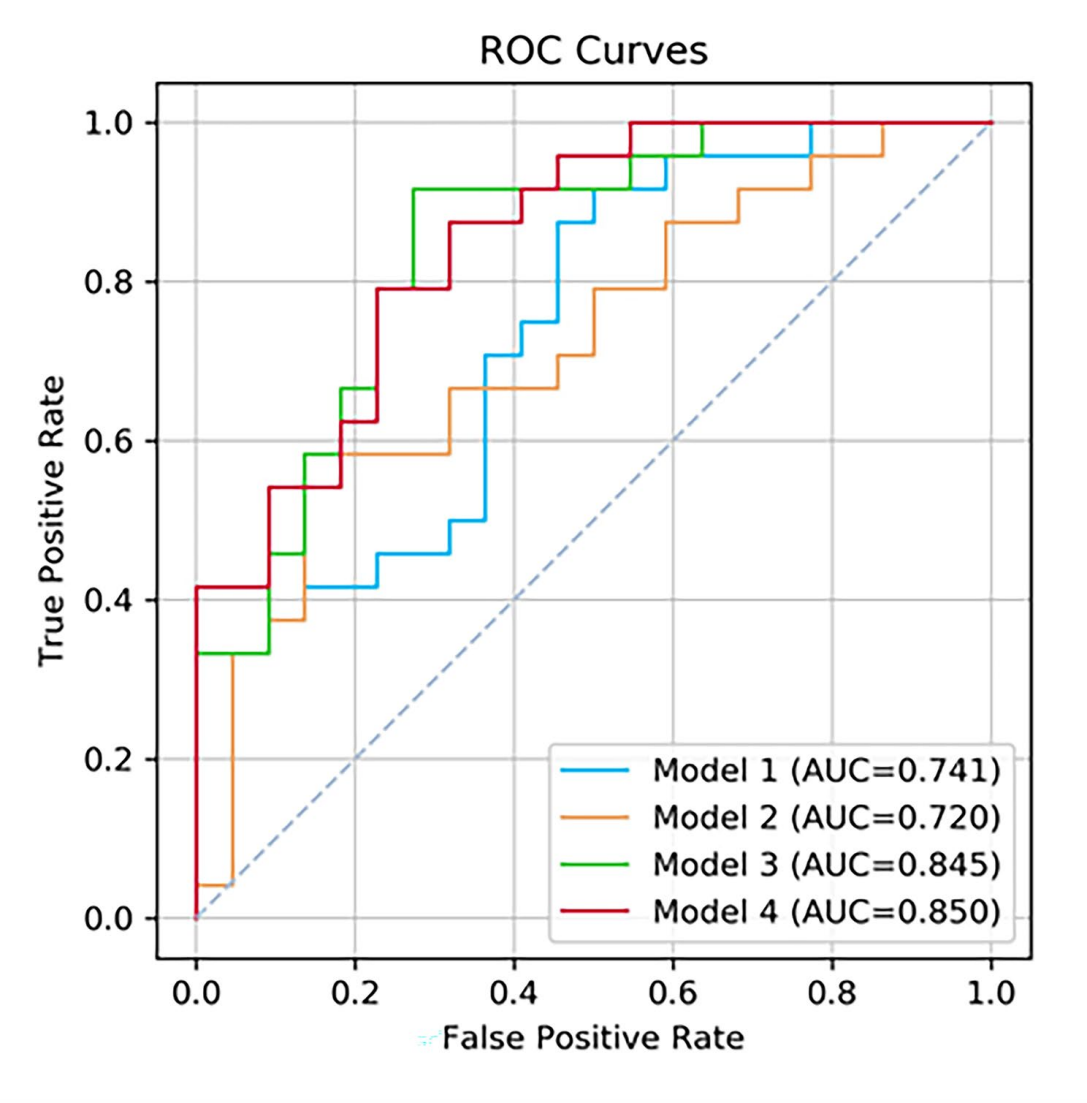
Receiver operating characteristic curves of the four models to classify pulmonary *A. baumannii* colonization and infection. Model 1, clinical baseline information + laboratory indicators and radiographic features of T_3_. Model 2, model1 + the change value of between T_3_ and T_1_. Model 3, model 1 + the change value of between T_3_ and T_2_. Model 4, model 2 + model 3. All statistical comparisons between area under the receiver operating characteristic curve values of models 1–4 were significant (*p* < 0.05)

We calculated the AUC, accuracy, sensitivity, and specificity of the various models for the classification of infection and colonization (Table 2; Fig. 2). The best-performing model was Model 4, with multiple time-series features and an AUC of 0.850(95%CI: 0.638–0.873). In the test set, the baseline model (Model 1, clinical baseline information + laboratory data and radiographic features of T_3_) had an AUC of 0.741(95%CI: 0.568–0.775), which was significantly improved by adding the change in value between T_3_ and T_2_ (Model 3, AUC = 0.845[95%CI: 0.680–0.875], *p* = 0.021). Adding multiple time-series features further improved the discriminatory power (Model 4, AUC = 0.850 vs. 0.741, *p* = 0.041). However, adding the change in value between T_3_ and T_1_ did not improve classification performance (AUC = 0.720[95%CI: 0.551–0.777] vs. 0.741[95%CI: 0.568–0.775], *p* = 0.743).

**Table 2:** The performance of differences models in the classification of infection and colonization

Model	AUC	Accuracy	Sensitivity	Specificity	*P* value
Model 1	0.741(0.568,0.775)	0.587(0.432,0.730)	0.458(0.256,0.672)	0.727(0.498,0.893)	0.001
Model 2	0.720(0.551,0.777)	0.652(0.498,0.786)	0.667(0.447,0.844)	0.636(0.407,0.828)	0.004
Model 3	0.845(0.680,0.875)	0.783(0.636,0.891)	0.833(0.626,0.953)	0.727(0.498,0.893)	<0.001
Model 4	0.850(0.638,0.873)	0.761(0.612,0.874)	0.833(0.626,0.953)	0.682(0.451,0.861)	<0.001

Note: Data in brackets are 95% CI. Model 1 (baseline model), clinical baseline information + laboratory data and radiographic features of T_3_; model 2, model 1 + the change value of between T_3_ and T_1_; model 3, model 1 + the change value of between T_3_ and T_2_; model 4 (multiple time-series model), model 2 + model 3. All statistical comparisons between the AUC values of models 1–3 were significant (*p* < 0.001). The best value(s) within each group are indicated with bold typeface

The multiple time-series model, which integrates temporal information from longitudinal chest radiographs, showed better performance in classification than single time-point information. In terms of the accuracy, sensitivity, and specificity of the model, when multiple time-series features were added, the accuracy and sensitivity were 17.4% (0.761, 95%CI: 0.621–0.874) and 37.5% (0.833, 95%CI: 0.626–0.953), respectively. Moreover, by additionally incorporating the change in value between T_3_ and T_2_, the accuracy improved by 19.6% (0.783; 95%CI: 0.636–0.891), and the sensitivity increased by 37.5% (0.833; 95%CI: 0.626–0.953). Additionally, the AUC of Model 3 was similar to that of model 4 (0.845 vs. 0.850, *p* = 0.912), whereas the accuracy and specificity of model 3 were higher than those of model 4 (0.783 vs. 0.761 and 0.727 vs. 0.682, respectively).

### Clinical use of time-series model to classify Infection and colonization

**Fig. 3:**
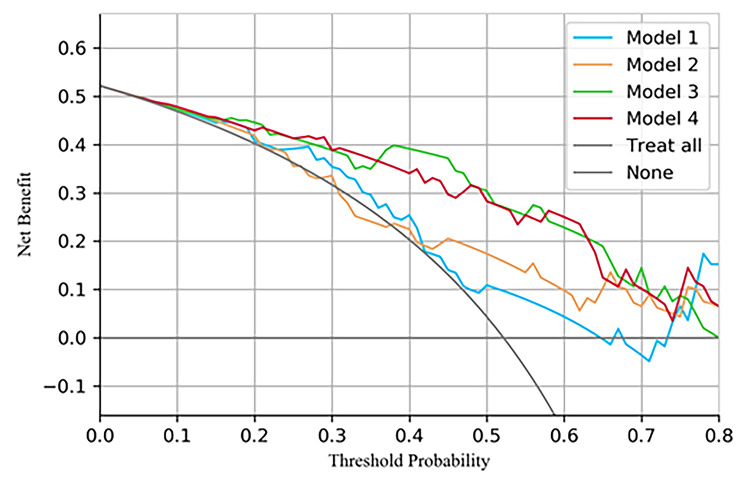
Decision curve analysis was performed to evaluate the net benefit, with the y-axis representing the net benefit. The optimal prediction to maximize net benefit was determined by identifying the higher curve at any given threshold probability. The results of the decision curve analysis demonstrated that the model utilizing all feature sets provided a greater net benefit compared to other models, highlighting its superior predictive performance

In terms of clinical utility value, decision curve analysis (DCA) showed that compared with the single time point clinical model, when the threshold probability ranged from approximately 0.10–0.75, the majority of patients benefited from Model 3 and Model 4, suggesting that the addition of the change in value between T_3_ and T_2_ or multiple time-series features provides a reliable clinical tool for predicting the status of pulmonary *A. baumannii*. The DCA based on these four models is shown in Fig. 3. Finally, we evaluated the calibration of the various models for classification. The predicted probabilities of model 2 and model 4 were close to the observed probabilities and showed good calibration (Figure S1).

### Feature importance

**Fig. 4:**
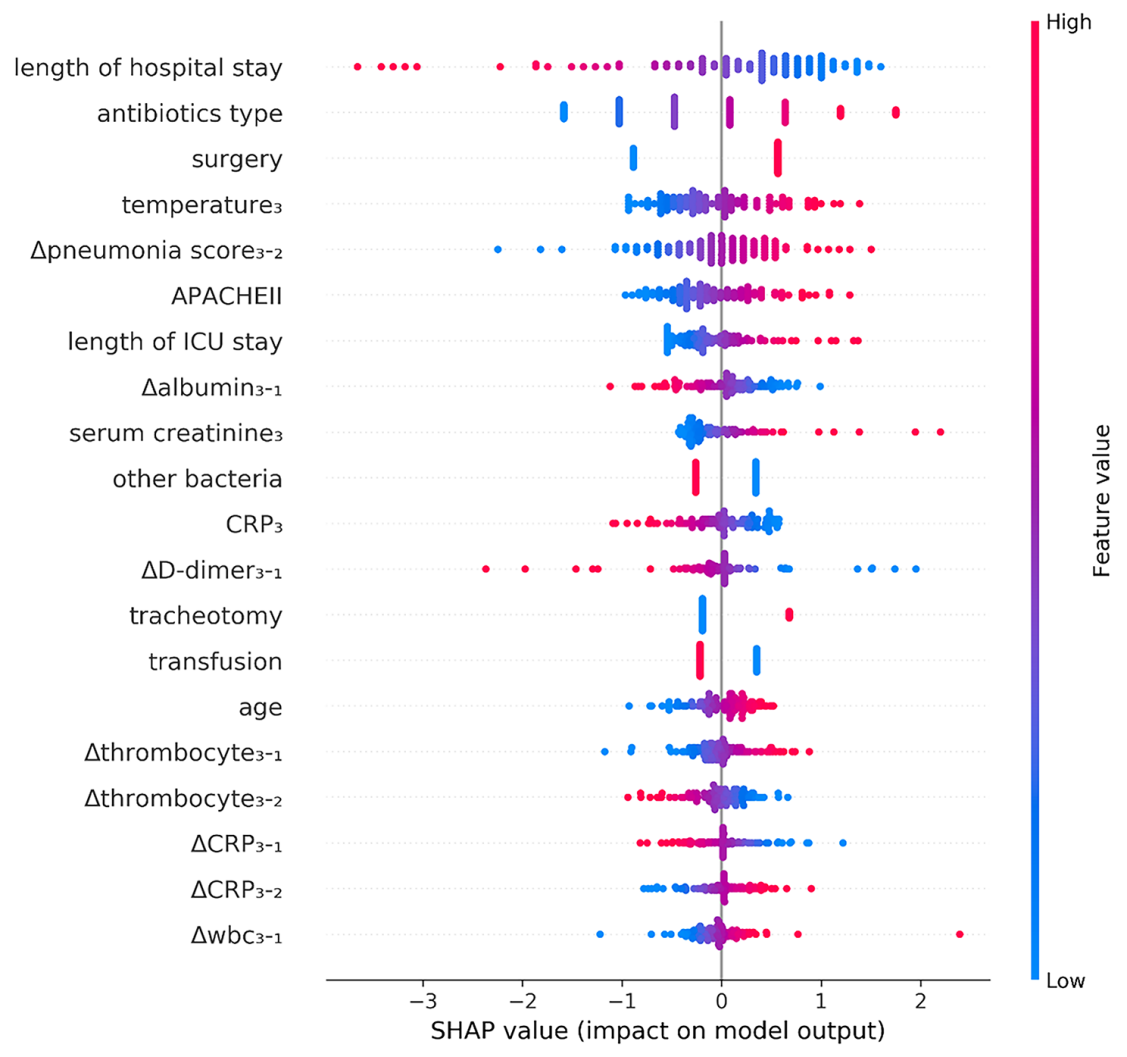
The SHAP summary plot illustrates 20 feature clusters, where the values of a specific feature (such as average, minimum, and maximum) are aggregated. The features are arranged in descending order on the y-axis, based on their mean absolute impact on the prediction. The SHAP value for each feature is represented by the distance of the dot from the x-axis at x = 0. A farther distance indicates a greater effect (positive or negative) that the feature had on the machine learning model’s output. The color of the dot corresponds to the original feature values, ranging from low (blue) to high (magenta), as indicated by the color bar

Figure 4 displays the characteristics sorted by the SHAP values for the best-performing model (model 4). Using SHAP analysis, we identified the 15 most informative features in our model. These features include the length of hospital stay, types of antibiotics used, surgical procedures after admission to hospital, temperature_3_, Δpneumonia score_3 − 2_, tracheotomy or not, ΔD-dimer_3 − 1_, APACHE II, Δalbumin_3 − 1_, combined culturing with other bacteria, CRP, length of hospital stay, Δ thrombocyte_3 − 2_, transfusion or not, serum creatinine_3_, Δthrombocyte_3 − 1_, ΔPCT_3 − 1_, ΔCRP_3 − 2_, age and hypoproteinemia. Intuitively, a longer length of hospitalization stay, length of ICU stay, higher Δ pneumonia score_3 − 2_, APACHE II, temperature, CRP, and the use of more types of antibiotics all contributed to a greater risk of infection, as predicted by the model. In particular, we found that radiographic changes contributed more to the model than changes in laboratory indicators.

**Fig. 5:**
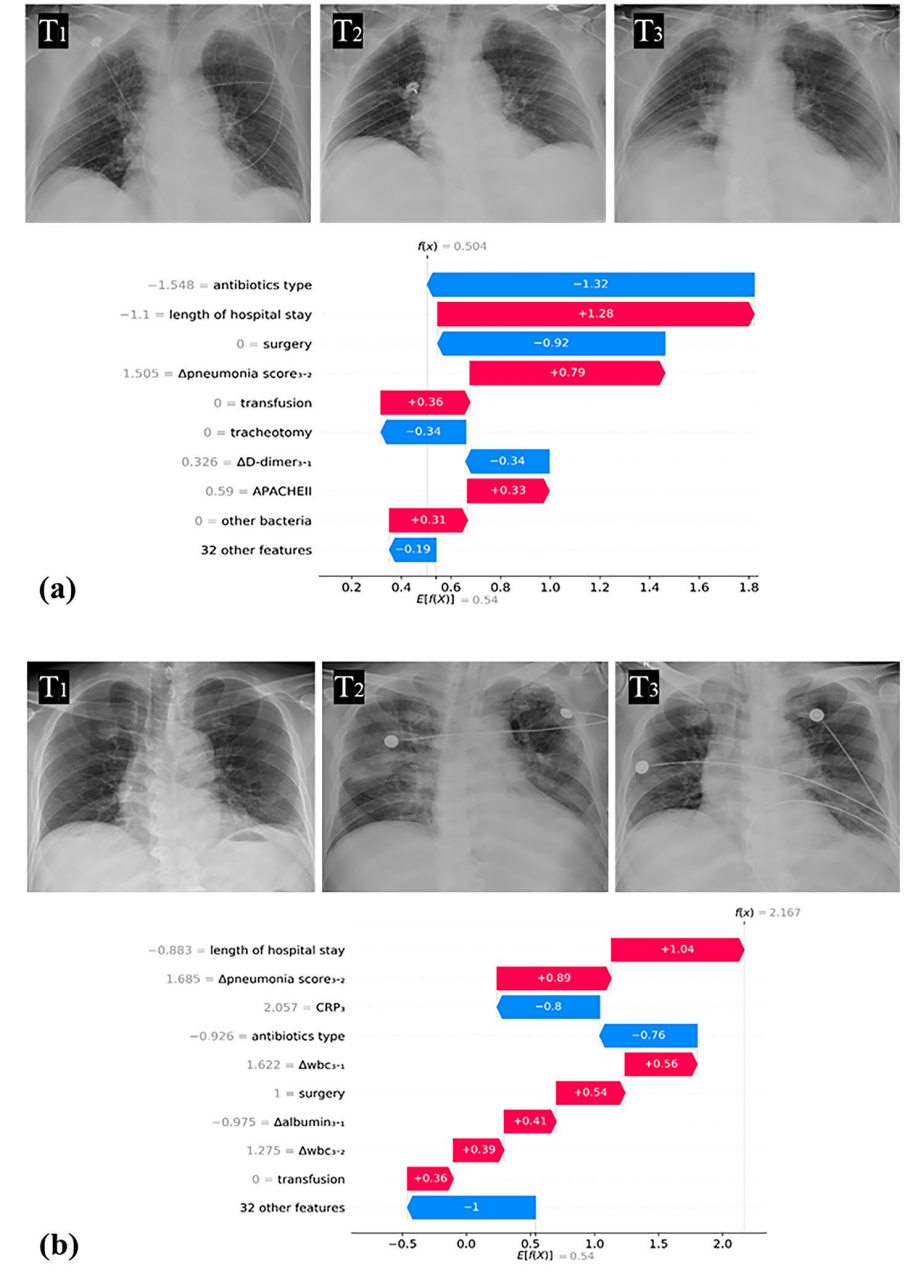
Two examples in the test set. On the left are the SHAP’s feature distribution analysis graphs of the highest predicted probability model prediction. The red bar represent the positive contribution to model’s prediction, while the blue bar means the negative contribution to its prediction. On the right are the radiographs of the cases. **(a)** An example of a 71-year-old female patient with intrahepatic bile duct stones with cholangitis. The strain was detected in the patient’s natural cough sputum 7 days after admission. The predicted probability of model 4 is 0.897. **(b)** A case with a 58-year-old female patient with glioblastoma of the frontal lobe. The strain was detected in the patient’s natural cough sputum 13 days after admission

In Fig. 5, the prediction outcomes of various models are depicted for two representative cases. The first is the case of infection that was misclassified as colonization by the baseline model but correctly classified by other time-series models. The second shows another example of colonization that was misclassified as infection by model 3 but correctly classified by model 4 (the multiple time-series model).

## Discussion

This study highlights the effectiveness of using machine learning algorithms to accurately classify pulmonary colonization and infection of *A. baumannii* by integrating clinical time-series imaging and laboratory characteristics. We incorporated nested models to investigate the predictive effectiveness of the models by integrating data on laboratory indicators and pneumonia scores at different time points. Among the different models tested, the multiple time-series model (model 4) showed the most promising results in terms of AUC, sensitivity, decision curve analysis, and calibration curve. Therefore, the multiple time-series model has the potential to be a valuable tool for identifying the pulmonary status of *A. baumannii*, aiding clinicians in making early adjustments to treatment regimens.

*A. baumannii* colonizes the respiratory tract by forming biofilms, leading to drug resistance and recurrent infections. This results in resistance to most antibacterial drugs and outbreaks [29]. Although colonization itself may not immediately cause infection, it can weaken the immune system and ultimately leading to infection. Our hypothesis is that changes occur in the patient’s microenvironment during this process, as indicated by fluctuations in inflammatory cell counts, imaging results, and other related indicators. One of the unique aspects of our model is that its inclusion of data not only at the time of the initial strain culture, but also at admission and 3 days before the first culture, while considering the changes in each index value. To capture the dynamic changes induced by *A. baumannii*, we developed a nested machine learning model based on laboratory data and chest radiographs at three different time points. As expected, our study demonstrates the additional value of dynamic change data for distinguishing between colonization and infection. The model’s expressive capability was enhanced with the addition of data from all three time points, resulting in improved performance. In comparison to models based on a single time point or two time points, the multiple time-series model achieved superior performance (AUC: 0.850 vs. 0.741 vs. 845 vs. 720), and exhibits high accuracy and sensitivity (0.761[95%CI: 0.612–0.874], 0.833[95%CI: 0.626–0.953], respectively). The top five dynamic variables identified by the model, which made significant contributions, namely Δpneumonia score_3 − 2_, Δalbumin_3 − 1_, ΔD-dimer_3 − 1_, Δthrombocyte_3 − 1_, and Δthrombocyte_3 − 2_.

In comparison to other lung infections, *A. baumannii*-induced lung changes exhibit atypical characteristics. To gain deeper insights into the potential patterns of lung alterations caused by *A. baumannii* and to further explore its diagnostic value in distinguishing between infection and colonization, we incorporated chest X-ray images from multiple time points. Through the analysis of these sequential X-ray images, our goal is to uncover the evolution and distinctive features of lung lesions resulting from *A. baumannii *infection. This comprehensive understanding will contribute to improved knowledge of the developmental dynamics associated with this infection, enabling clinicians to establish a more accurate foundation for diagnosis and treatment. Pneumonia was defined as the presence of new or progressive pulmonary infiltrates on chest radiographs [18]. For hospitalized patients, particularly those in the ICU, routine chest radiography is common. Our findings demonstrated that one imaging metric emerged as one of the top five contributors to the overall prediction accuracy of the model. Specifically, the most significant imaging factor for classification was the difference in chest radiograph pneumonia scores between the day of the initial culture of the strain and 3 days before culture. We observed that pneumonia in infected patients progressed within 1 day of the strain culture, in contrast to 3 days before culture, where the colonization group showed a reduction in pneumonia, with a statistically significant difference. This result implies that the increase in the chest radiograph pneumonia score from 3 days prior to the culture of the strain to the culture of the strain could serve as a more indicative sign of infection. This information is currently concealed within the trends observed for chest radiograph pneumonia. By promptly detecting early warning signs, we can effectively differentiate between colonization and infection and administer early treatment to infected patients. This approach holds the potential to provide timely assistance and improve patient outcomes.

SHAP values were applied to determine which features contributed the most to model predictions in the logistic regression predictions and calculate the degree of contribution of each significant feature to the model. The higher accuracy of multiple time-series models can be attributed to the inclusion of additional distinct variables, especially laboratory values such as the difference in albumin levels between T_3_ and T_1_, D-dimer, thrombocytes, CRP, and WBC count. These findings highlight the untapped potential of using multiple time-point laboratory data as potential biomarkers for precise classification of infection and colonization.

Regarding clinical applicability, the multiple time-series model (model 4) has the potential to guide individual therapies against *A. baumannii* from cultured sputum or alveolar lavage fluid. For patients with infections, the therapy should be targeted based on drug sensitivity results, to reduce the multiplication of bacteria in the body. In this study, 21 of 24 (87%) infected patients who could benefit from adjusting treatment regimens were successfully identified using multiple time-series model.

Our multiple time-series model holds great potential in assisting clinicians, particularly young doctors, surgeons, and intensivists, in identifying the status of *A. baumannii* in the lungs. Additionally, the model will play a significant role in preventing and controlling nosocomial infections, making it an important criterion for clinical research enrollment. However, this study has several limitations. Firstly, the quantitative analysis of pneumonia scores was subjective and not computer-assisted, which may introduce potential bias. Additionally, the current study is limited to a retrospective analysis conducted at a single center with a relatively small sample size. Therefore, conducting a larger-scale study is necessary to validate the clinical utility and generalizability of the model before its widespread application in clinical practice. Future research should consider expanding the study to multiple centers and increasing the sample size to ensure greater accuracy and reliability of the model’s results. Collaborating with other medical institutions in conducting prospective studies across different clinical settings would provide a comprehensive evaluation of the model’s applicability. Through rigorous validation and clinical application, we can gain a better understanding of the model’s clinical efficacy. By means of additional validation, we can enhance our comprehension of the clinical usefulness of the model, and furnish physicians with more practical and convenient decision support for identifying *A. baumannii *status and implementing measures for nosocomial infection prevention and control.

## Conclusion

In conclusion, the proposed model, incorporating time-series chest radiographs and laboratory data, shows promise in the early detection of *A. baumannii* infection and colonization. We envision a potential implementation strategy in clinical practice. For high-risk patients in respiratory and critical care, neurosurgery, and critical care medicine departments, regular chest radiographs and monitoring of laboratory indicators should be conducted, with close attention to any dynamic changes in the results. In the presence of detected *A. baumannii* strains, the model can provide risk estimation and recommend personalized treatment strategies. However, further large-scale prospective studies are necessary to validate the effectiveness of this approach.

### Electronic supplementary material

Below is the link to the electronic supplementary material.

Supplementary Material 1

## Data Availability

The datasets used and/or analysed during the current study are available from the corresponding author on reasonable request.
