# Supplementary material for: Using time-series chest radiographs and laboratory data by machine learning for identifying pulmonary infection and colonization of Acinetobacter baumannii
Source: Respir Res. 2024 Jan 3;25:2. doi: 10.1186/s12931-023-02624-x (PMC10765646; doi:10.1186/s12931-023-02624-x)
Supplement: Supplementary file 1 — Supplementary Material 1 [file 12931_2023_2624_MOESM1_ESM.docx]

**Assembly of Cases and Controls**

We conducted a search on the case system of Nanfang Hospital of Southern Medical University for patients with *Acinetobacter baumannii (A. baumannii)* cultured from the respiratory tract between 2018 and 2020. These cases were carefully analyzed by a team of respiratory and infection specialists, who collaborated to assess the strain's presence in the body based on established Consensus guidelines. Patients with ambiguous results that could not be clearly classified were excluded from the study. Subsequently, patients were screened according to predefined inclusion and exclusion criteria, resulting in 152 patients who met the eligibility criteria. Inclusion and exclusion criteria were as follows:

Inclusion criteria:

1.Patients with *A. baumannii* cultured in sputum or alveolar lavage fluid more than two consecutive times.

2. Patients who can be clinically identified as colonized or infected.

Exclusion criteria:

1. Patients with lower respiratory tract chest abnormalities resulting from non-infectious causes such as pulmonary embolism, pulmonary edema, lung cancer, and other conditions.

2. Patients with strains cultured within three days of hospitalization.

3. Patients who do not undergo routine chest radiographs.

**Data collection**

There are 61 features in this study including multiple time point change values as follows: (1) Demographic characteristics: ages and sex; (2) Preoperative comorbidities: hypertension, diabetes, anemia, coronary heart disease, cardiac insufficiency, atrial fibrillation, hypoproteinemia, respiratory failure, chronic obstructive pulmonary disease, bronchiectasis, cerebrovascular disease; (3) Invasive procedures: urinary catheterization, endotracheal intubation, tracheotomy, nasal catheter, bronchoscope, central venous catheter, urinary catheter, and surgical procedures; (4) Use of drugs before culturing out the strain: types of glucocorticoid and antibiotics; (5) Hospitalization before culturing out the strain: length of hospitalization and intensive-care units (ICU); (6)Time-series data (including three phases: T_1_, within 1 day of admission; T_2_, 3 days before culturing out the strain; T_3_, 1 day within culturing out the strain) : chest radiographs, serum inflammatory indicators, liver function, kidney function, and electrolytes.

For data with multiple points in time we try to present it using the change value approach. Table E1 provides a summary of demographic, clinical and time-series characteristics in infection and colonization groups.

**Criteria for distinguishing between colonization and infection**

*A. baumannii* infection is a common occurrence in critically ill patients and is frequently linked with other bacterial and/or fungal infections. When *A. baumannii* is detected in sputum or alveolar lavage, a medically experienced physician will assess whether it indicates colonization or infection based on the Consensus of the Chinese specialists for diagnosis, treatment, and control of *A. baumannii* infection. The following factors are taken into consideration: in addition to general signs of bacterial infection, such as fever, elevated white blood cell and neutrophil counts, and C-reactive protein, the following factors should be taken into account to determine *A. baumannii* lung infection: (1) Clinical symptoms, signs, and new, persistent, or aggravated pulmonary exudates, infiltrates, and solid changes on imaging consistent with pneumonia. (2) Host factors, including underlying disease, immune status, prior antimicrobial use, and other risk factors associated with disease development, such as duration of mechanical ventilation. (3) The emergence of *A. baumannii* in a patient receiving antimicrobial therapy, who had shown improvement for a period and then worsened in a timeframe consistent with the appearance of *A. baumannii*. (4) Clinical significance of positive culture results in terms of specimen collection method, specimen quality, bacterial concentration (quantitative or semiquantitative cultures), and smear findings. (5) Two or more sputum cultures showing pure *A. baumannii* growth or dominant *A. baumannii* growth. It is essential to consider these criteria when determining the clinical significance of *A. baumannii* isolation.

**Imaging Analysis: Quantification of Chest Radiograph Abnormalities**

We adopt a two-person independent reading approach, wherein each reader reads randomly for different patients. However, for the same patient, the reading order follows the sequence of the first, second, and third time points. All reading is done through the Picture Archiving and Communication System (PACS) system, and the viewer can adjust the window width and position to read the images. And it was scored according to Table E2. After independent evaluation, the radiologists resolved any disagreement with discussion and consensus.

**Figure E1: The Calibration curve of model 2 and model 4**

**Table E1: Comparison of Demographic, Clinical, Time-series Laboratory data and Imaging Characteristics between Groups**

**Table E2: Chest X-ray Pneumonia Scoring Criteria**

**Figure E1**


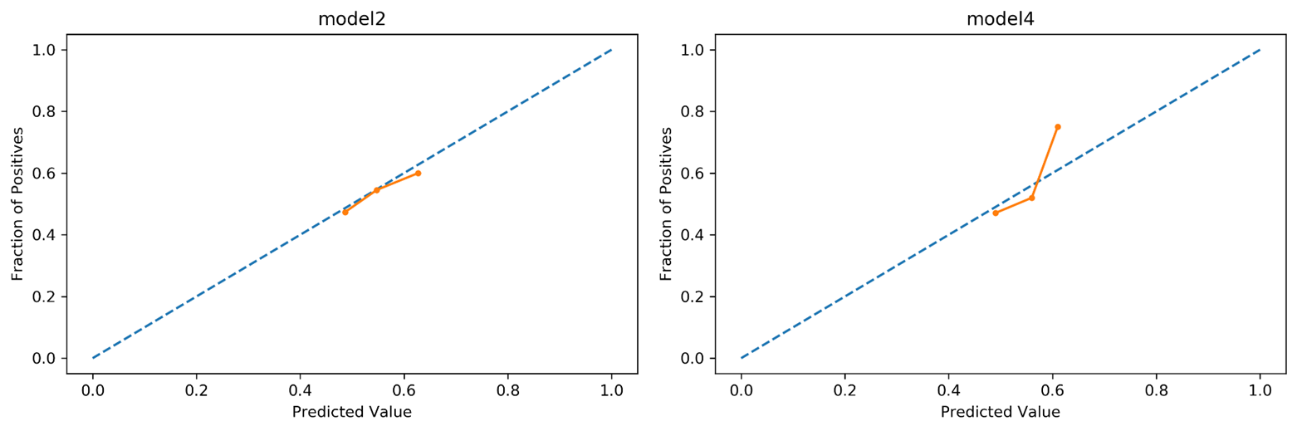


**Table E1**

| Characteristic | Infection  (n=80) | Colonization  (n=72) | *Ρ* value |
| --- | --- | --- | --- |
| Age (y), median (IQR) | 66(53-74) | 62(51-70) | 0.173 |
| Comorbidities, n (%) |  |  |  |
| Hypertension | 37(46.3) | 30(41.7) | 0.570 |
| Diabetes | 15(18.8) | 17(23.6) | 0.463 |
| Anemia | 31(38.8) | 24(33.3) | 0.488 |
| Coronary heart disease | 13(16.3) | 11(15.3) | 0.870 |
| Cardiac insufficiency | 26(32.5) | 17(23.6) | 0.224 |
| Atrial fibrillation | 11(13.8) | 5(6.9) | 0.172 |
| Hypoproteinemia | 48(60.0) | 31(43.1) | **0.037** |
| Respiratory failure | 30(37.5) | 19(26.4) | 0.143 |
| Chronic pulmonary disease | 12(15.0) | 5(6.9) | 0.116 |
| Bronchiectasis | 5(6.3) | 2(2.8) | 0.308 |
| Cerebrovascular disease | 12(15.0) | 3(4.2) | **0.025** |
| ICU, n (%) | 71(88.8) | 65(90.3) | 0.759 |
| Previous surgical procedures, n (%) ^ƚ^ | 49(61.3) | 46(63.9) | 0.737 |
| Transfusion, n (%) ^ƚ^ | 45(56.3) | 45(62.5) | 0.434 |
| Cancer, n (%) | 19(23.8) | 17(23.6) | 0.984 |
| Combined other bacteria, n (%) ^ƚ^ | 38(47.5) | 38(52.8) | 0.516 |
| Combined fungal, n (%) ^ƚ^ | 23(28.7) | 8(11.1) | **0.007** |
| Urinary catheter, n (%) ^ƚ^ | 71(88.8) | 66(91.7) | 0.547 |
| CVC (d) ^ƚ^ | 11.79±1.15 | 11.14±1.25 | 0.702 |
| Bronchoscope, (times) ^ƚ^ | 0.93±0.30 | 0.92±0.25 | 0.979 |
| ICU, (times) ^ƚ^ | 1.03±0.53 | 1.11±0.07 |  |
| Length of hospital stay (d) | 14.29±0.97 | 16.44±1.36 | 0.192 |
| Length of ICU stay(d) | 11.03±1.11 | 9.28±0.85 | 0.214 |
| Duration of Oxygen supply (d) ^ƚ^ | 0.93±0.20 | 0.92±0.25 |  |
| Endotracheal intubation | 6.00±0.713 | 6.47±0.97 | 0.691 |
| Tracheotomy | 3.18±0.97 | 2.57±0.76 | 0.631 |
| Nasal catheter | 2.08±0.47 | 1.74±0.63 | 0.663 |
| Types of drugs, median (IQR) ^ƚ^ |  |  |  |
| Glucocorticoid | 2(1-2) | 2(1-2) | 0.208 |
| Antibiotics | 4(2-5) | 3(2-4) | **0.031** |
| APACHEII, median (IQR) | 14(10-19) | 12(8-16) | **0.006** |
| Temperature_3_(℃) | 37.64±0.099 | 37.28±0.094 | **0.009** |
| Pneumonia score_3_ | 15.60±0.88 | 13.61±0.86 | 0.108 |
| Laboratory results^§^ |  |  |  |
| Percentage of neutrophils (%) | 81.18±1.07 | 81.41±1.08 | 0.880 |
| CRP (mg/L) | 80.99±6.17 | 77.33±7.17 | 0.702 |
| D-dimer (mg/mL) | 7.03±0.87 | 8.94±1.53 | 0.260 |
| WBC (×10^9^/L) | 13.23±1.03 | 11.00±0.52 | 0.055 |
| PCT (ng/ml) | 1.77±0.46 | 1.20±0.23 | 0.307 |
| Serum creatinine (μmol/L) | 122.92±12.29 | 97.47±10.91 | 0.127 |
| Albumin (g/L) | 34.72±0.68 | 35.36±0.63 | 0.493 |
| Hemoglobin (g/L) | 96.61±4.60 | 95.28±2.36 | 0.797 |
| thrombocyte (×10^9^/L) | 228.35±12.87 | 242.45±15.17 | 0.477 |
| Δvalue_3-2_ |  |  |  |
| ΔCRP_3-2_ | 3.27±7.37 | -16.56±11.00 | 0.124 |
| ΔWBC_3-2_ | 1.92±1.00 | -0.55±0.79 | 0.055 |
| ΔPCT_3-2_ | -0.74±0.66 | -2.96±1.24 | 0.100 |
| ΔD-dmier _3-2_ | 0.10±0.93 | -1.63±1.65 | 0.348 |
| ΔAlbumin _3-2_ | -3.97±4.58 | 2.18±0.77 | 0.215 |
| ΔSerum creatinine _3-2_ | 10.81±7.84 | -13.45±9.08 | **0.044** |
| ΔPercentage of neutrophils _3-2_ | 2.047±1.49 | 1.84±1.80 | 0.929 |
| ΔHemoglobin _3-2_ | 1.07±4.49 | -2.64±1.83 | 0.466 |
| ΔThrombocyte _3-2_ | 19.14±9.53 | 29.25±13.28 | 0.531 |
| ΔPneumonia score _3-2_ | 0.51±0.62 | -2.75±0.70 | **0.001** |
| Δvalue_3-1_ |  |  |  |
| ΔCRP_3-1_ | -1.04±12.06 | 13.11±11.44 | 0.401 |
| ΔWBC_3-1_ | 2.87±1.12 | 0.58±0.81 | 0.097 |
| ΔPCT_3-1_ | -5.15±2.83 | -4.37±3.32 | 0.860 |
| ΔD-dimer _3-1_ | -2.48±1.75 | 1.20±2.40 | 0.207 |
| ΔAlbumin _3-1_ | 0.004±0.86 | 0.057±0.90 | 0.966 |
| ΔSerum creatinine _3-1_ | -5.41±11.90 | -22.55±7.78 | 0.247 |
| ΔPercentage of neutrophils_3-1_ | 3.63±1.83 | 7.47±2.63 | 0.224 |
| ΔHemoglobin _3-1_ | -19.73±4.90 | 7.47±2.63 | 0.910 |
| ΔThrombocyte _3-1_ | 23.29±13.59 | 8.86±20.18 | 0.546 |
| ΔPneumonia score _3-1_ | 4.08±0.94 | 1.84±1.15 | 0.131 |

Note. Except where indicated, data are means ± SDs. ^ƚ^ Indicates the current period of hospitalisation. ^§^ Baseline data is the data within 1 day of culturing out the strain. The symbol "Δ" represents the value of change between the data at different time points. Δvalue _3-2_ represents the change value of the time-series feature between T3 and T2. Δvalue _3-1_ represents the change value of the time-series feature between T3 and T1. Bolded indicates statistically significant differences. CVC= central venous catheter, CRP=C-reactive protein level, PCT=procalcitonin, WBC=white blood cell

**Table E2**

| Area |  | Grading standard |
| --- | --- | --- |
| Lung | Upper lung field | GGO, consolidation (Based on the affected area):  0, involvement;  1, less than 5%;  2, 5%-25%;  3, 25%–50% involvement;  4, 50%–75% involvement;  5, greater than 75% involvement. |
|  | Middle lung field |  |
|  | Lower lung field |  |
| Pleural effusion | | 0, no involvement;  1, small amount of pleural effusion;  2, large pleural effusion. |
| Pleural thickening adhesions | | 0, involvement;  2, no involvement. |
| Total points: 34 points. | | |
